# Social Determinants of Remaining Life Expectancy at Age 60: A District-Level Analysis in Germany

**DOI:** 10.3390/ijerph19031530

**Published:** 2022-01-29

**Authors:** Achim Siegel, Jonas F. Schug, Monika A. Rieger

**Affiliations:** Institute of Occupational and Social Medicine and Health Services Research, University Hospital Tübingen, Wilhelmstr. 27, 72074 Tübingen, Germany; jonas-friedrich.schug@student.uni-tuebingen.de (J.F.S.); monika.rieger@med.uni-tuebingen.de (M.A.R.)

**Keywords:** population health, life expectancy, life expectancy at older age, social determinants of health, inequalities in health, health inequity

## Abstract

Remaining life expectancy at age 60 (in short: RLE) is an important indicator of the health status of a population’s elders. Until now, RLE has not been thoroughly investigated at the district level in Germany. In this study we analyzed, based on recent publicly available data (2015–2017), and for men and women separately, how large the RLE differences were in Germany across the 401 districts. Furthermore, we examined a wide range of potential social determinants in terms of their bivariate and multivariate (i.e., partial) impact on men’s and women’s RLE. Men’s district-level RLE ranged between 19.89 and 24.32 years, women’s district-level RLE between 23.67 and 27.16 years. The best single predictor both for men’s and women’s RLE at district level was ‘proportion of employees with academic degree’ with standardized partial regression coefficients of 0.42 (men) and 0.51 (women). Second and third in rank were classic economic predictors, such as ‘household income’ (men), ‘proportion of elder with financial elder support’ (women), and ‘unemployment’ (men and women). Indicators expressing the availability of medical services and staffing levels of nursing homes and services had at best a marginal partial impact. This study contributes to the growing body of evidence that a population’s educational level is a decisive determinant of population health resp. life expectancy in contemporary industrialized societies.

## 1. Introduction

Life expectancy at birth in a given population is often used—whether implicitly or explicitly—as a summary or proxy variable for the health status of a population: the higher life expectancy at birth, the better the health status of the respective population as a whole [1,2,3,4,5,6,7]. This is easily comprehensible and quite ‘intuitive’ if one considers that life expectancy at birth—but also life expectancy at higher ages, e.g., at the age of 60—is usually calculated on the basis of period life tables containing a population’s age-specific mortality probabilities: to determine life expectancy at birth, these age-specific mortality probabilities are added up sequentially, starting with age 0 (newborns) and progressing sequentially to the next higher age until the sum of these probabilities reaches 0.50 [6,7,8,9,10,11]. Usually, the age-specific mortality probabilities are based on the mortality data of three consecutive calendar years (e.g., 2015–2017). If one considers this method of calculating life expectancy, it becomes clear that life expectancy as an indicator of a population’s health status can comprehensively trace the impact of, e.g., wars and other social disasters like the influenza pandemic in 1918–2020 on the concerned societies in history [12,13], improvements in economic conditions [14,15], specific developments in medical knowledge and innovations in the health care system based on these developments [16,17,18,19,20], of political developments [21,22] or currently of the direct and indirect impact of the COVID-19 pandemic (2020–2021) on life expectancy in contemporary societies [23,24,25,26,27,28,29,30].

In Germany, like in many other high-income countries (e.g., [31,32,33,34,35,36]), life expectancy at birth differs markedly among different parts of the country, i.e., among federal states, regions and districts [1,2,8,37,38]. A recent study on district-level life expectancy in Germany [2] demonstrated that average life expectancy at birth varied among Germany’s 401 districts by 5.3 years for men and 3.9 years for women, based on the period life tables of 2015/17. Although the differences in district-level life expectancy have narrowed since the period immediately following the reunification of Germany in 1990—when men’s life expectancy at birth varied by more than 8 years among districts (e.g., 8.5 years in 1997/99 [7]), the differences found in recent studies still appear considerable, especially in view of the fact that an official political goal in Germany is to create “equivalent living conditions” in the whole of the federal territory [39,40]. This goal is so important that Germany’s constitution (cf. Article 72, Para. 2) explicitly grants the federal government the right to intervene in the policies of the federal states when “necessary in the national interest” [41]. In addition, the German Social Code V, § 70, requires health insurance funds and health care providers to provide “needs-based and equitable care for insured persons in accordance with the generally accepted state of medical knowledge” [42].

The above-mentioned recent study on district-level life expectancy at birth in Germany [2] also investigated, in an explorative manner, a series of potential social determinants to explain the differences in life expectancy among the 401 districts. Thus, for example, the authors examined the influence of general indicators such as ‘gross domestic product (GDP) per capita’ in the individual districts or ‘physician density’ (primary-care physicians per 100,000 inhabitants) as an indicator to operationalize access to primary health care in a district. Above all, the authors tested the influence of economic indicators that addressed the economic living conditions of the socially and economically weakest groups in society: they examined the influence of unemployment, child poverty, the proportion of people receiving housing subsidies and so-called Hartz-IV welfare benefits, as well as the proportion of elder inhabitants receiving the government’s financial elder support [2]. Similar to earlier studies in Germany on this issue [1] and to international studies (cf. below), the authors of this more recent study [2] registered an outstanding explanatory power of economic indicators, especially those that focused on the living conditions of the economically weakest inhabitants: unemployment, Hartz-IV support, and child poverty had the greatest explanatory power in statistical terms regarding both men’s and women’s district-level life expectancy at birth, with standardized bivariate regression coefficients ranging between −0.5 and −0.6 each in case of men’s life expectancy at birth and amounting up to −0.4 in case of women’s life expectancy at birth [2], p. 496f.

Many other studies on high-income and developing countries have come to similar results: indicators mapping social or economic inequalities (such as unequal distribution of income, unemployment or the proportion of people dependent on social welfare support) have usually been among the strongest of the investigated predictors for life expectancy at birth or all-cause mortality (e.g., [38,43,44,45,46,47,48,49,50,51,52]).

Comparable to the importance of life expectancy at birth as a measure of overall population health, remaining life expectancy at age 60 or 65 is a common indicator of population health at older ages in international studies (cf., [33,46,48,53,54,55,56,57]). However, only few studies have systematically examined remaining life expectancy at age 60 (or 65) for its variation by region or district and with respect to its social determinants within a country [33]. For Germany with its political obligation to establish “equivalent living conditions” in the federal territory (cf. above), a study investigating remaining district-level life expectancy at old age—analogous to the above-mentioned studies of Rau and Schmertmann [2] or Latzitis et al. [1] on life expectancy at birth—has not yet been conducted. We aim to close this research gap with this publication. In this article we want to answer the following three exploratory research questions:

(1) How large are the differences in district-level remaining life expectancy for 60-year-old men and women in Germany?
(2) Is there a specific geographic distribution pattern of areas with high or low remaining life expectancy in Germany (both among men and women)? How similar are the spatial distribution patterns of men’s and women’s remaining life expectancy at age 60?
(3) Which social determinants explain the existing differences in district-level remaining life expectancy of 60-year-old men and women in statistical terms?

## 2. Data and Methods

### 2.1. Data

To answer the three research questions, we first of all relied on data provided by the Federal Institute for Research on Building, Urban Affairs and Spatial Development (in German: Bundesinstitut für Bau-, Stadt- und Raumforschung, in short BBSR) [58]; this institute belongs to the Federal Office for Building and Regional Planning (BBR) [59]. The BBSR provides an “interactive online atlas” comprising hundreds of spatial indicators to characterize living conditions in Germany and Europe. This online database is called INKAR (short for “Indikatoren und Karten zur Raum- und Stadtentwicklung”), which means indicators and maps for spatial and urban development [60].

The INKAR database does not only provide data on life expectancy at birth from the federal to the district level, but also on remaining life expectancy at age 60 (abbreviated from here as RLE) both for men and women down to the district level. These life expectancy data were calculated from period life tables of three consecutive years according to the method of William Farr ([61], p. 44f). These period life tables were based on age-specific mortality data of the 401 German districts for the three years 2015–2017 on the one hand and on population data (inhabitants) for each district for the age groups 60–64, 65–69, 70–74, 75–79, 80–84, and 85+ on the other hand; the data on the number of inhabitants per district had been extrapolated from the 2011 census ([61], p. 44f). Furthermore, INKAR contains data on important social and economic indicators (federal to district level, some even to community level) that have often been referred to as social determinants of life expectancy such as educational level of a population, GDP per capita, household income, unemployment and unemployment rate, proportion of inhabitants receiving social welfare payments of different kind, voter turnout in different elections, but also data on the availability of health services (such as density of primary-care physicians or density of hospital beds); cf. the list of available indicators in [61].

As a supplementary data source, we used “Regionaldatenbank Deutschland”, edited by the German Statistical Offices [62], to get data on district-level availability of certain social services we thought were important but were not contained in the INKAR database (cf. below).

To analyze men’s and women’s RLE at district level, we used the most recent data provided by INKAR; these data had been derived from population and mortality data of 2015, 2016, and 2017 (in short: 2015/17).

### 2.2. Methods to Investigate the Research Questions

To answer research questions (1) and (2), we picked remaining life expectancy values at age 60 for each district directly from the INKAR database. Using interactive INKAR tools, RLE values for the various districts were grouped in quintiles. The resulting spatial distribution of remaining life expectancy quintiles was then visualized by a map of Germany in which the various districts were colored according to which particular quintile they belonged to. The RLE values for the three districts with the highest and for the three districts with the lowest values were reported individually. The similarity of the spatial distribution pattern of ‘remaining life expectancy at age 60′ for men with the distribution pattern regarding women was measured by Pearson’s correlation coefficient.

To answer research question (3), we followed a two-step procedure: in the first step we conducted bivariate regression analyses with district-level RLE (for men and women separately) as the dependent variable and selected social, political, economic and health services indicators as independent variables (cf. below). For each bivariate regression model, we reported standardized regression coefficients (ß values, including corresponding *p* values) as a measure indicating the effect size of the predictor’s impact on RLE.

In a second step, we conducted multiple regression analyses with RLE as the dependent variable (separately for men and women). All variables that had been checked as potential predictors in the bivariate analyses were included as independent variables in the multivariate models unless they were inter-correlated to a very high degree (r ≥ 0.80) with other predictor variables [63]. In the latter case, only those predictors were kept for the multivariate model which were correlated more strongly with the dependent variable (i.e., with RLE) in the bivariate analyses. To exclude statistically insignificant predictors and thus to select the final multiple regression model, we used the method of backward elimination of independent variables (criterion: probability of F value for elimination > 0.050). For the final regression models, F values (and the respective *p* values), and corrected R^2^ values were determined for the regression models as a whole, while standardized regression coefficients (ß values) and their respective *p* values were determined for the statistically significant predictors. In this exploratory study we limited our multivariate data analysis to the standard multiple regression procedure, as just described. We did not use more complex methods, such as geographically weighted regression (e.g., [64]), in order to keep the results as simple as possible and comparable with similar studies on the topic [1,33].

Corresponding to Cohen’s thumb rule, determination coefficients (R^2^ values) from 0.02 to 0.12 were interpreted as indicative of a small amount of explained variance, from 0.13 to 0.25 as indicative of a moderate amount and from 0.26 as representing a high amount of explained variance ([65], p. 413ff). Following Cohen’s thumb rule for the interpretation of Pearson’s correlation coefficients (r values) as well as standardized regression coefficients (ß values), values amounting from 0.10 to 0.29 were regarded as indicating a small effect, from 0.30 to 0.49 a moderate effect, and from 0.50 a large effect ([65], p. 77ff).

Data were analyzed with the statistics software IBM SPSS Statistics for Windows, version 28 [66].

### 2.3. Potential Social Determinants of Men’s and Women’s Remaining Life Expectancy at Age 60 at District Level

To investigate the impact of potential social determinants of men’s and women’s RLE at district level, we chose the following 12 potential determinants from the INKAR and Regionaldatenbank Deutschland databases [60,62] (Table 1):

These indicators are based on data for the year 2017 except indicator (2) which is based on data of 2016 [61]. All indicators were taken from INKAR [60] except indicators (3) and (4) which were extracted from Regionaldatenbank Deutschland [62,67].

Some remarks on why we selected the above-mentioned potential determinants seem to be appropriate at this point. In principal, we concentrated on social and economic factors that are considered as the strongest and most consistent predictors of population health or life expectancy such as education, average household income, unemployment, proportion of people dependent on social welfare support), cf., [2,68,69]. These factors are embodied here by indicators (5)–(11). Beyond this, we were interested in how strong the impact of important features of the health and social services system (indicators (1)–(4)) was on the health status of the elder, i.e., on RLE, in comparison to the social and economic predictors just mentioned. Furthermore—and in general—we looked for indicators that had been used (and thus “tested”) at least in the German context as predictors of a population’s health status or life expectancy or were used as ‘components’ when researchers developed a multidimensional deprivation index for Germany; we outline this briefly in the following:

Indicators (1) and (2) reflect inhabitants’ access to important health services, whereas indicators (3) and (4) illustrate the staffing levels of two essential care services that primarily serve the elder. These four indicators had also been considered by an earlier German study in which predictors of district-level life expectancy at birth had been investigated [1]; indicator (1) had been investigated as a predictor by a more recent study in which the impact of socioeconomic indicators was compared with the impact of primary-care physician density on life expectancy at birth in the 401 districts of Germany [2].

Indicators (5) and (6) measure the overall economic strength of a district’s population, with ‘GDP per capita’ representing the ‘GDP production’ aspect and ‘disposable household income’ operationalizing the use of the GDP in form of end consumers’ income; both of these indicators were also used in the study of Latzitis et al. [1], and one of these two indicators—GDP per capita—in the more recent study by Rau and Schmertmann [2].

Indicators (7) and (8) represent two different indicators of employees’ education at district level. These two variables stand for the educational resp. vocational level of a district’s population; indicator (7) is used as one of several indicators making up the “German Index of Multiple Deprivation” [70,71,72], whereas indicator (8) is a component of the “German Index of Socioeconomic Deprivation” [73].

While indicators (9) and (10) reflect a district’s employment deprivation and income deprivation at working age respectively, indicator (11) can be interpreted as a measure of income deprivation at retirement age. All of these three indicators were investigated as predictors by the above-mentioned recent German study on district-level life expectancy at birth [2]; moreover, indicator (9) is a component of both above-mentioned multidimensional deprivation indices [70,71,72,73].

The last indicator in the list (12), voter turnout in federal elections, is regarded as a measure for elementary political participation of a district’s population. In several international studies, considerable associations between populations’ voter turnout and health status have been found [74,75,76,77,78,79,80], and some authors—including German ones—conceive voter turnout as a proxy variable for ‘social capital’ and thus a predictor of a population’s health status [70,71,72,74,78,80,81]—a procedure which has been thoroughly analyzed in the past [81,82,83].

## 3. Results

The following two Sections (Section 3.1 and Section 3.2) provide results to answer research questions (1) and (2), while Section 3.3 and Section 3.4 provide results to answer research question (3).

### 3.1. Remaining Life Expectancy at Age 60 in Germany and in German Districts (2015/17)

The average remaining life expectancy at age 60 (in short: RLE) among men in the whole of Germany amounted to 21.90 years (based on period life tables 2015/17); the RLE among women in Germany was about 3.5 years higher: it amounted to 25.45 years (based on period life tables 2015/17) [58].

Men’s district-level RLE ranged from 19.89 years (district with minimum RLE) to 24.32 years (district with maximum RLE); this means that the average RLE for men differed among the 401 districts by up to 4.43 years. As to women’s district-level RLE, the range was considerably smaller: women’s district-level RLE ranged from 23.67 years to 27.16 years—i.e., the difference amounted up to 3.49 years.

As to men’s RLE, the three districts with the highest RLE were Starnberg, federal state Bavaria (24.32 years), Breisgau-Hochschwarzwald, federal state Baden-Württemberg (24.09 years) and Fürstenfeldbruck, federal state Bavaria (24.03 years). The three districts with men’s lowest RLE in Germany were Herne, federal state North Rhine-Westphalia (19.89 years), Gelsenkirchen, federal state North Rhine-Westphalia (19.93 years) and Sonneberg, federal state Thuringia (19.95 years).

Regarding women’s RLE, the three districts with the highest RLE were Dresden, federal state Saxony (27.16 years), Starnberg, federal state Bavaria (26.91 years) and Munich City, federal state Bavaria (26.88 years). The three districts with women’s lowest RLE were Flensburg, federal state Schleswig-Holstein (23.67 years), Bremerhaven, federal state Bremen (23.84 years), and Solingen, federal state North Rhine-Westphalia (23.98 years).

### 3.2. Geographical Distribution of RLE in German Districts (2015/17), Grouped in RLE Quintiles

**Figure 1:**
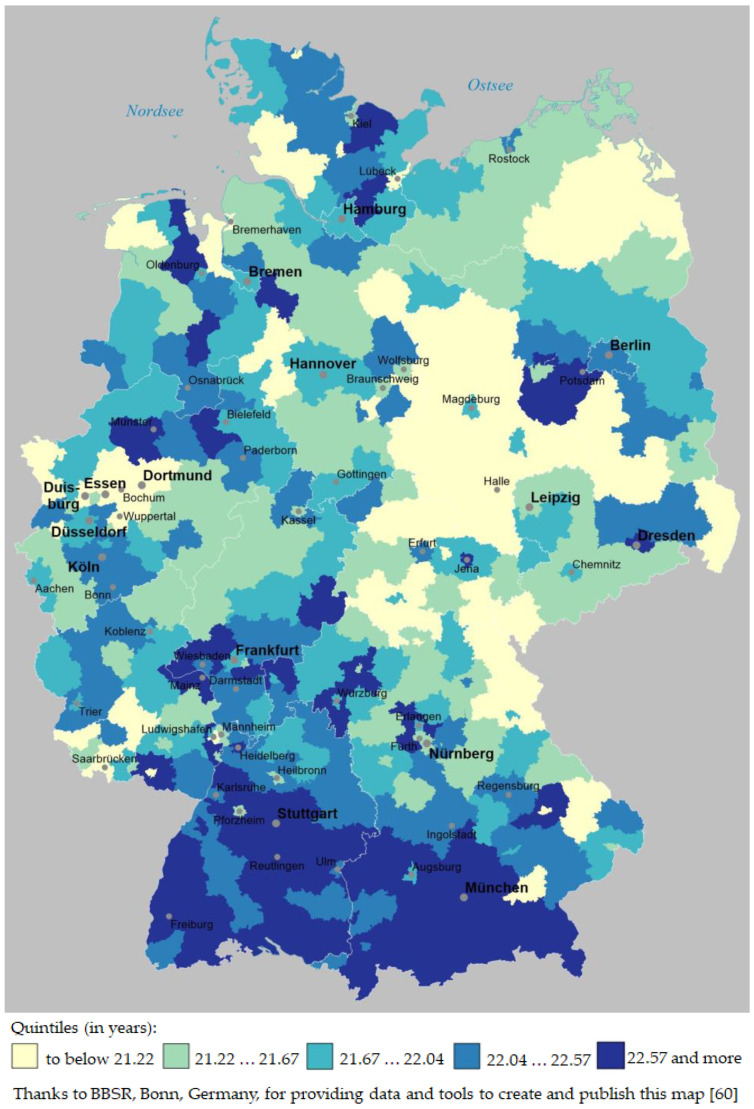
Men’s remaining life expectancy at age 60 at district level in Germany, grouped in quintiles and based on period life tables 2015/17.

The geographical distribution of men’s RLE, grouped in quintiles, is shown in Figure 1.

Figure 1 shows that large areas of Southern Germany belong to the highest RLE quintile (22.57 years and more); these areas cover large parts of the federal states Baden-Württemberg (around Stuttgart, Reutlingen and Freiburg) and the southern parts of Bavaria (around Munich). On the other hand, large areas belonging to the lowest quintile (lower than 21.22 years) can be found in Eastern Germany—particularly in the federal states Saxony-Anhalt and Thuringia (around Magdeburg, Halle, and Erfurt)—but also in the very densely populated Ruhr region in North Rhine-Westphalia (around Duisburg, Essen, and Dortmund).

**Figure 2:**
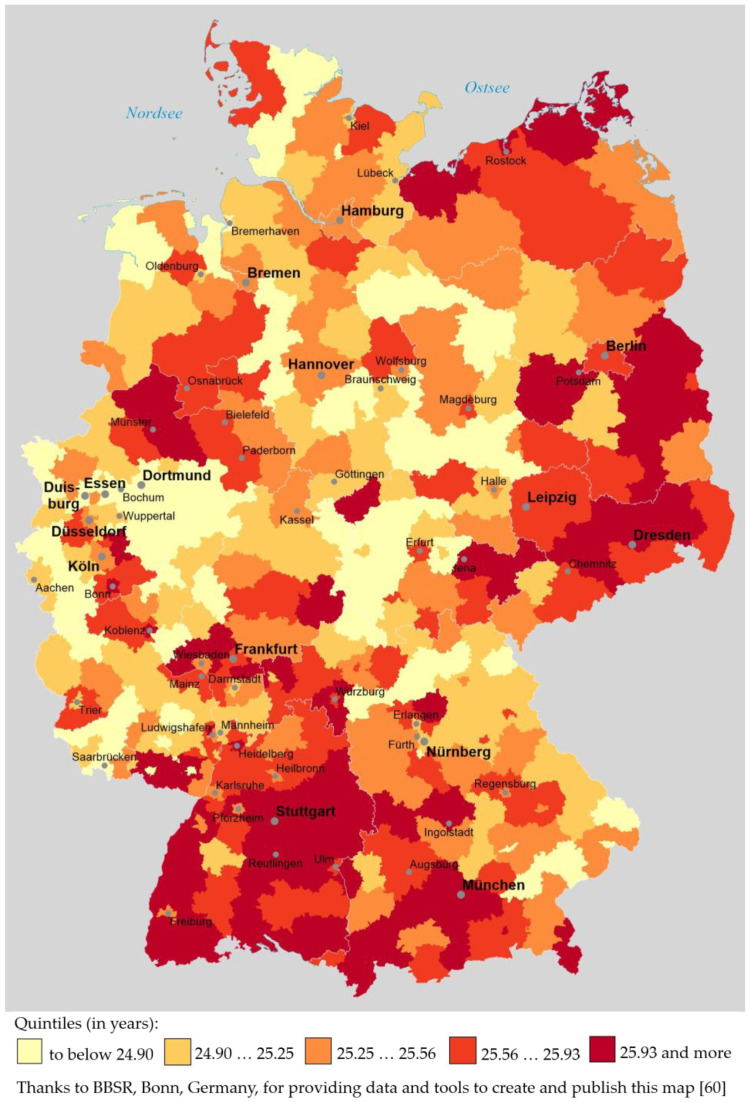
Women’s remaining life expectancy at age 60 at district level in Germany, grouped in quintiles and based on period life tables 2015/17.

The geographical distribution of women’s RLE, grouped in quintiles, is shown in Figure 2.

Figure 2 shows that in southern Germany, particularly in the southwestern federal state Baden-Württemberg and in southwestern Bavaria (around Munich), there are large areas belonging to the highest RLE quintile (25.93 years and more). However, unlike to men’s RLE distribution, there are also large areas in Eastern Germany that belong to the highest RLE quintile (around Dresden, Jena or east of Berlin). Large areas belonging to the lowest RLE quintile (lower than 24.89 years) can not only be found in Western Thuringia (west of Erfurt) and in Southern Saxony-Anhalt (south of Magdeburg), but also in the Ruhr region and the Sauerland region in North Rhine-Westphalia (around Duisburg, Essen, Dortmund and south of Dortmund). All in all, the disparity between Eastern and Western Germany that can still be found regarding men’s RLE (cf., Figure 1) is no longer present when regarding women’s RLE. One should not forget, though, that the range of district-level RLE is about one year smaller in case of women than in case of men (cf. above).

Thus, the similarity between the distribution patterns regarding men’s RLE vs. women’s RLE seems fairly high, but not extremely high; this visual impression is confirmed by a Pearson’s correlation coefficient of r = 0.69; *p* < 0.001.

These differences between men’s and women’s RLE distribution patterns add plausibility to a systematical differentiation between men’s and women’s RLE when analyzing social determinants as potential predictors for RLE.

### 3.3. Analyzing Potential Predictors of Men’s and Women’s Remaining Life Expectancy at Age 60 Using Bivariate Regression Analyses

The indicators which were used as predictors in the regression models were presented above in Table 1 (Section 2.3).

In Table 2 we present the results of the bivariate regression analyses with men’s RLE as the dependent variable and the above-mentioned indicators (Table 1) as predictors.

Table 2 shows that economic indicators are indeed among the best bivariate predictors: household income (ß = 0.63), unemployment (ß = −0.60) and proportion of people with Hartz-IV support (ß = −0.56) rank among the best four predictors. The predictor with the highest ß value is, however, a non-economic indicator: voter turnout (ß = 0.67). The indicator ‘proportion of elder with financial elder support’, expressing income deprivation at retirement age, is only weakly correlated with the dependent variable (ß = −0.10). The two indicators reflecting district-level availability of medical services (i.e., the first two indicators in Table 2) are weakly negatively associated with men’s RLE at district level, whereas the two indicators depicting the staffing level of (outpatient or inpatient) nursing services for the elder are weakly positively associated with the target variable (ß = 0.14 and ß = 0.22).

In Table 3 the results of the bivariate regression analyses with women’s RLE as the dependent variable are presented.

Table 3 shows that most indicators listed here are not associated as highly with women’s RLE as with men’s RLE (cf., Table 2). First of all, the standardized regression coefficients of indicators expressing social or economic inequalities do not exceed a ß value (amount) of 0.40. Furthermore, the predictive power of ‘voter turnout’ is lower here (ß = 0.38) than in case of men’s RLE (ß = 0.67; cf., Table 2). As in case of men’s RLE, the four indicators expressing the availability of medical services or the staffing level of nursing services for the elder are consistently weakly correlated with women’s RLE, with ‘care personnel per 100 persons in need of outpatient care services’ still having the highest beta amount (ß = 0.14).

An analysis of social predictors of men’s and women’s remaining life expectancy at the age of 60 would not be complete if we omitted a multivariate analysis. The results of the multivariate analyses are presented in Section 3.4.

### 3.4. Analyzing Potential Predictors of Men’s and Women’s Remaining Life Expectancy at Age 60 Using Multivariate Regression Analyses

In the multiple regression analysis of men’s and women’s RLE at district level, 11 (out of 12) indicators could be checked as potential predictors. The indicator ‘proportion of people with Hartz-IV support’ was intercorrelated almost perfectly (r = 0.95) with ‘unemployment’ and was therefore excluded from the multiple regression according to the procedural rules described in Section 2.2.

The resulting multiple regression model with men’s district-level RLE as the target variable explained the target variable to a statistically significant degree: F (7, 393) = 84.124; *p* < 0.001; the amount of explained variance of men’s RLE at district level was high (corrected R^2^ = 0.59). The results regarding the individual predictors in that model are presented in Table 4.

In the multiple regression model with men’s RLE as the target variable, the indicator ‘proportion of employees with academic degree’ had the greatest partial impact (ß = 0.42). A considerably weaker partial association turned out for ‘household income’ (ß = 0.27) and ‘unemployment’ (ß = −0.23). Still weaker, but statistically significant partial associations resulted for ‘care personnel per 100 persons in need of full inpatient care’ (ß = 0.16), ‘voter turnout’ (ß = 0.15), ‘GDP per capita’ (ß = −0.13) and ‘proportion of elder with financial elder support’ (ß = −0.09).

As in the multiple regression analysis of men’s RLE at district level, 11 indicators were checked as potential predictors in the multiple regression analysis of women’s RLE at district level. The resulting multiple regression model was significant: F (3, 397) = 17.398; *p* < 0.001), and the amount of explained variance can be regarded as high (corrected R^2^ = 0.34). The results for the individual predictors are presented in Table 5.

As can be seen from Table 5, the best predictor for women’s RLE at district level was ‘proportion of employees with academic degree’ (ß = 0.51). Second in rank was ‘proportion of elder with financial elder support’ (ß = −0.30), followed by ‘unemployment’ (ß = −0.23). No other predictors had a statistically significant partial impact on the target variable, including those indicators expressing availability of medical services and staffing level of nursing services for the elder.

## 4. Discussion

### 4.1. Discussion of the Results That Provide Answers to Research Questions (1) and (2)

In Section 3.1 we presented data that provide the answer to the first research question (“How large are the differences in district-level remaining life expectancy for 60-year-old men and women in Germany?”): the range of RLE across the 401 districts in Germany amounted to 4.43 years for men (minimum: 19.89 years; maximum: 24.32 years) and 3.49 years for women (minimum: 23.67 years; maximum: 27.16 years). These ranges are almost as high as in the case of men’s and women’s life expectancy at birth: based on period life tables 2015/17, the range of district-level life expectancy at birth for men was recently estimated to 5.33 years (75.92–81.15 years) and to 3.92 years for women (81.77–85.69 years) [2]. Thus, the range in case of district-level life expectancy at the age of 60 for men resp. women amounted to 83% (4.43/5.33 years) resp. 89% (3.49/3.92 years) of the range of men’s and women’s respective district-level life expectancy at birth. This means that the greatest part of the life-expectancy-at-birth range at district level can be attributed to a difference in mortality rates of inhabitants from the age of 60 years. In other words: health inequity (measured by life expectancy at district level) in contemporary Germany crystallizes quite clearly when life expectancy is operationalized as remaining life expectancy at older ages. Thus it comes to no surprise at all that the well-known ‘gender gap in life expectancy’ [84,85,86,87] can also be found when regarding remaining life expectancy at older ages.

In Section 3.2 we analyzed data that provide answers to the second research question (“Is there a specific geographic distribution pattern of areas with high or low remaining life expectancy in Germany, both among men and women? How similar are the spatial distribution patterns of men’s and women’s remaining life expectancy at age 60?”): the analysis demonstrated that the spatial distribution pattern of areas with high vs. low RLE was similar among men and women, but not extremely similar (r = 0.69; cf. Section 3.2): for men, a difference between former East and West Germany (i.e., between the former German Democratic Republic and the Federal Republic of Germany) could still be seen on the map, with RLE being higher in districts belonging to the former West Germany (cf. Figure 1); in case of women’s RLE, no such East vs. West Germany distribution pattern was visible (cf. Figure 2). Nevertheless, regarding the geographic distribution of RLE at district level, there are still no “equal” or “equivalent living conditions” on German territory [39,40] as there are still—or again—regions that consistently stand out positively (southern Baden-Württemberg, southwestern Bavaria) but also regions that have well below-average RLE scores for both men and women (such as, e.g., the Ruhr region in the federal state North Rhine-Westphalia and some regions in eastern Germany).

### 4.2. Discussion of the Results That Provide Answers to Research Question (3)

In Section 3.3 and Section 3.4 we presented results that enable us to answer the third research question (“Which social determinants explain the existing differences in district-level remaining life expectancy of 60-year-old men and women in statistical terms?”). To investigate the impact of potential determinants of men’s and women’s RLE at district level, we chose 12 potential determinants from the ‘INKAR’ and ‘Regionaldatenbank Deutschland’ databases [60,62]; these 12 determinants were characterized in greater detail in Section 2.3, Table 1.

Our investigation of the third research question was based on a two-step procedure: (a) a selection of 12 potential predictors to depict the most important social and economic predictors and compare their relative impact with that of health and social services indicators that should be particularly important for the elder, (b) a series of bivariate regression models with men’s and women’s RLE as target variables and 12 potential predictors each, and (c) two multiple regression analyses with men’s and women’s RLE as target variables and 11 predictors each. In the following, we briefly discuss each of these three procedures.

#### 4.2.1. Selection of the Potential Predictors of Remaining Life Expectancy at Old Age

In the ‘social determinants of health’ research community, there is—until now—no agreed model or taxonomy regarding which factors are the most influential or consistent social determinants of the health status or life expectancy of a (certain type of) population [68,88]. Until now there is not even an agreed criterion as to what should be considered a social determinant of health [88]. In view of these theoretical and conceptual inconsistencies in the field, we stuck in our study, as outlined in Section 2.3, to mapping those predictors that have been conceived and/or demonstrated in numerous studies to be the most influential and consistent determinants: educational level, income and other socio-economic variables focusing on the living conditions of the most deprived groups in society (e.g., [2,3,4,38,43,44,45,47,51,53,68,69,89]). These aspects were mapped in this study by predictors (5)–(11) (cf. Table 1), as outlined in Section 2.3. above. These seven indicators included also two indicators reflecting different aspects of the educational level of a population (indicators (7) and (8) in Table 1); remarkably, no indicators focusing on educational level were investigated in the recent study of Rau and Schmertmann [2].

In addition to predictors (5)–(11), we selected four predictors to depict important characteristics of the health and social services system in the German context, with particular regard to the needs of the elder. The first of these four indicators had been investigated also by Rau and Schmertmann [2]. Going beyond Rau and Schmertmann’s predictors, we examined indicators (2)–(4) (cf. Table 1) as additional potential predictors because we wanted to assess the impact of the health and social services system on RLE more broadly. Indicators (3) and (4) depict social services that primarily serve the elder population, i.e., the target group of this study. Similarly, indicator (11) (cf. Table 1) can be considered as a predictor indicating poverty specifically among the elder population—this predictor was also examined by Rau and Schmertmann [2]. Contrary to Rau and Schmertmann, we omitted the indicator ‘child poverty’ ([61], p. 68) because it did not seem to be a sufficiently specific indicator for our target group.

Finally, we additionally explored the predictive status of indicator (12), i.e., ‘voter turnout’, as a proxy variable indicating the amount of ‘social capital’ of a district’s population; with this operationalization we followed several other authors of previous studies [70,72,79,90,91]. Though being a component of the ‘German Index of Multiple Deprivation’ [70,71,72], this indicator had—to the best of our knowledge—not been examined as a predictor of its own in the German context.

#### 4.2.2. Discussion of the Results of the Bivariate Regression Models

Similar to Rau’s and Schmertmann’s bivariate analysis of eight social and economic determinants of district-level life expectancy at birth in Germany [2], predictors reflecting social and economic inequalities in society were better predictors in our study than indicators expressing availability of medical services and staffing levels of care services for the elder (i.e., than predictors (1)–(4) in our study, cf. Table 1). The best three predictors in the study of Rau and Schmertmann were ‘unemployment’, ‘proportion of people with Hartz-IV support’ and ‘child poverty’ (the latter had not been investigated in our study) for both men’s and women’s life expectancy at birth, amounting to ß values between −0.5 and −0.6 each for men and about −0.4 each for women [2]. Furthermore, the bivariate effects of these social and economic determinants on life expectancy were consistently higher for men than for women—a pattern that was also found in our bivariate analyses (cf. above, Table 2 and Table 3). Similar to Rau’s and Schmertmann’s analyses of life expectancy at birth, ‘GDP per capita’ and ‘proportion of elder with financial elder support’ also had only small effects on both men’s and women’s RLE in our bivariate analyses. Regarding the results of the study of Rau and Schmertmann, then, our bivariate results seem roughly in line with the former, although we investigated remaining life expectancy at age 60 (and not life expectancy at birth).

In our bivariate analyses, however, two other indicators stood out as having strong or moderate effects on the outcome variables—two indicators that do not belong to the domains of economic or health services indicators and thus were not analyzed by Rau and Schmertmann: first, ‘voter turnout’ (indicating ‘social capital’) reached ß values of 0.67 (men’s RLE) and 0.38 (women’s RLE); second, ‘proportion of employees with academic degree’ turned out as a predictor with a moderate effect size for both men’s and women’s RLE (ß = 0.41 for men; ß = 0.40 for women).

#### 4.2.3. Discussion of the Multiple Regression Models

Applying the ‘backward elimination’ method for selecting the final multiple regression models for men’s and women’s RLE at district level, some important similarities for men and women resulted:

(i) In both final models, ‘proportion of employees with academic degree’ turned out as the best partial predictor for the respective target variables, amounting to ß = 0.42 for men’s RLE and 0.51 for women’s RLE at district level (cf. Table 4 and Table 5).
(ii) Only second and third in rank were classic economic predictors: in case of men’s RLE, these indicators were ‘household income’ (ß = 0.27) and ‘unemployment’ (ß = −0.23) and in case of women’s RLE ‘proportion of elder with financial elder support’ (ß = −0.30) and ‘unemployment’ (ß = −0.23).
(iii) Furthermore, variables reflecting the availability of health services or the staffing level of care services for the elder—i.e., indicators (1)–(4) in Table 1—had an at best marginal partial impact on the outcome variables: in case of men’s RLE, only ‘care personnel per 100 persons in need of full inpatient care’ had a very small positive impact (ß = 0.16); in case of women’s RLE, none of those four indicators had a statistically significant partial impact on the target variable.
(iv) Finally, for both men’s and women’s RLE at district level, ‘voter turnout’ had a far smaller association in the multiple regression models than in the bivariate regression models: with regard to men’s RLE, its ß value decreased from 0.67 in the bivariate to 0.15 in the multivariate analysis; for women’s RLE, there was not even a significant partial impact of ‘voter turnout’ in the multiple regression model, whereas in the bivariate model it had been 0.38.

What stands out as a difference between men’s and women’s multiple regression results is that ‘proportion of elder with financial elder support’ plays a considerably greater role for women’s than for men’s RLE. This can be attributed to the fact that in Germany women are considerably more affected by old-age poverty than men because women’s employment biographies were (and still are) interrupted more often than those of men; therefore and because women’s salary is on average lower than that of men in Germany, women‘s pensions are—as a rule—much smaller than those of men [92,93]. This disparity cannot, as a rule, be compensated by financial elder support. For that reason ‘proportion of elder with financial elder support’ is considered an indicator for ‘old-age poverty’ (‘Altersarmut’ in German) [61].

All in all, the similarities between the results for men’s RLE in comparison to those for women’s RLE seem considerably greater in light of the multivariate analyses than on the grounds of the bivariate models.

Until now there is, to the best of our knowledge, only one international study that investigated a comparably wide range of potential area-level predictors of remaining life expectancy at old age: the study of Laborde et al. in France [33], which investigated district-level remaining life expectancy (and moreover remaining disability-free life expectancy) at age 60. France, as Germany’s neighbor, should rather well compare to Germany because of a roughly similar social and economic structure, educational attainment levels and development of medical and social services. In Laborde et al.’s multivariate regression analysis of remaining life expectancy at age 60, the ratio of “manual workers” in relation to “higher-level occupations” in an area (in a French “départment”) was the best predictor for both men’s and women’s RLE. According to the authors, this variable was to reflect “the occupational structure of the départment, placing emphasis on under- or over-representation of unskilled jobs with potentially more detrimental working conditions as compared to highly qualified jobs”. While there is no exact correspondence between this variable and any of the predictors examined in our study, the similarity between that variable and ‘our’ predictor ‘proportion of employees with academic degree’ seems obvious. If one accepts this argument, the corresponding result of Laborde et al.’s study might be interpreted as an analogue to our result mentioned above as (i). (Further variables reflecting the educational level of an area were not investigated by Laborde et al.) Somewhat less significant were (in Laborde et al.’s study) the predictors ‘unemployment’ and ‘proportion of the population living in large urban areas’ which were both negatively associated with RLE at départment level for men and for women. Five predictors reflecting the density of medical and nursing services in an area were still less significant or not at all significant in the multivariate analyses. Thus, the results of Laborde et al. on RLE at age 60 seem to be, in rough terms, similar to the results of our study; this adds to the plausibility of the results of our study.

### 4.3. Strengths and Limitations of This Study

In this exploratory study we examined a set of 12 potential predictors which were selected on the one hand on theoretical grounds, in that those 12 determinants reflected the impact of various kinds of social and economic inequalities such as differences in educational level, income and other socio-economic variables, differences in the level of medical and social services and elementary political participation as a proxy variable for the ‘social capital’ of a district. On the other hand, the 12 selected potential predictors had been used in various previous studies as social determinants of life expectancy at birth or as ‘components’ of deprivation indices (cf. Section 2.3) and thus could be considered as ‘tested’ social predictors of health.

Another strength of our study seems to be that we conducted not only bivariate analyses (as some studies on area-level life expectancy did), but also multivariate analyses of the target variables. The multivariate analyses made it possible to put the results of the bivariate analyses into perspective and to consider them in the context of other potential determinants. Thus, e.g., the high resp. moderate effect size of ‘voter turnout’ in the bivariate analyses was eliminated or at least considerably reduced on the grounds of the multivariate analyses. And only multiple regression models revealed that the predictor structure for men’s and women’s RLE at district level was fairly similar.

Two important limitations of this study result from how the target variables ‘remaining life expectancy at age 60′ (for men and women separately) were constructed. The target variables were derived from the population and death statistics in German districts; this could have resulted in two different kinds of bias. First, the 401 German districts differ considerably in size: the most populated district, Berlin City, had more than three million inhabitants at the end of 2017, whereas the smallest district—Zweibrücken, located in the federal state of Rhineland-Palatinate—had only about 34,000 inhabitants at the same time [2]. The median district size was about 150,000 inhabitants in 2017 [2]. Thus, it is obvious that the RLE estimates of small districts are much more prone to short-term fluctuations than RLE estimates of bigger districts; this might be a possible source for biased data regarding small districts. Second, RLE estimates for individual districts can be influenced by selective migration patterns [2]. Thus, for example, the migration of academically educated, healthy and wealthy retirees from below-average regions (e.g., the Ruhr region in North Rhine-Westphalia) to better-off districts like Starnberg (federal state of Bavaria) or to other regions in Southwestern Bavaria or Southern Baden-Württemberg would decrease RLE in their home district and increase RLE in their new district. Such selective migration phenomena could indeed be demonstrated for some regions in Europe [94]. The influence of such phenomena on the target variables can, however, neither be detected nor accounted for in our analyses.

A further limitation of the study is that we analyzed only a limited number of predictors for the target variables. Thus, it may be—despite prior analysis of the literature on the topic—that we did not consider important predictors for the target variables.

Furthermore, in checking the results of the bivariate analysis between predictor and target variables, we limited ourselves to ordinary multiple regression analyses, leaving aside more complex methods such as, e.g., geographically weighted regression (cf. Section 2.2). It cannot be ruled out that the results would differ if we had employed such a more complex method.

Moreover, a general methodological limitation of the study is that we ultimately cannot rule out reverse causality between predictor and target variables.

## 5. Conclusions

This is the first study that examined remaining life expectancy at age 60 and its social determinants at district level in Germany. RLE at district level in Germany ranged, according to period life tables 2015/17, between 19.89 and 24.32 years for men and between 23.67 and 27.16 years for women. These ranges (4.43 years for men, 3.49 years for women) seem fairly high, particularly in view of Germany’s constitutional claim to guarantee “equal” or “equivalent living conditions” on the whole of the German territory (cf. above, Section ‘Introduction’). Obviously, living conditions in German districts are not equitable and have not been equitable during the last decades, neither regarding life expectancy at birth [1,2,7] nor regarding remaining life expectancy at age 60.

The educational level of a district, measured by ‘proportion of employees with academic degree’, turned out as the best single predictor of both men’s and women’s RLE at district level, displaying standardized partial regression coefficients of ß = 0.42 (men) and ß = 0.51 (women). Second and third in rank were classic economic predictors such as, e.g., ‘proportion of elder with financial elder support’ or ‘unemployment’. In contrast, the availability of medical services and the staffing levels of nursing homes and outpatient nursing services for the elder had only an at best marginal impact both on men’s and women’s RLE at district level. Our results are in line with other studies focusing on life expectancy at birth in Germany insofar as the availability of medical services [2] and the staffing levels of nursing homes and nursing services for the elder [1] had only—if at all—a marginal impact on (remaining) life expectancy at district level. Thus, one may conclude at least that there seems to be no compelling evidence that district-level disparities (regarding the availability of basic medical services or staffing levels of care services for the elder) have a substantial impact on population health at district level.

The results of this study contribute to the growing body of evidence that a population’s educational level is a decisive—if not the decisive—determinant of population health resp. life expectancy, at least when regarding developed industrialized societies [95,96,97,98,99,100,101,102,103,104,105].

With respect to future area-level analyses of life expectancy at birth as well as remaining life expectancy at older ages we recommend to check the results of bivariate analyses in any case by multivariate analyses. Furthermore, future analyses of social determinants of area-level life expectancy (whether life expectancy at birth or remaining life expectancy at older ages) should include not only indicators that reflect the availability or quality of medical and nursing services or classic economic predictors (including those that express economic inequalities in society) but also indicators that specifically reflect the educational level of populations. Indicators of this latter kind seem to have been neglected too often in previous studies. In this spirit the authors of a recent study on rising life expectancy in three Western industrial states during the last 30 years concluded with regard to the role of education: “In addition to all the other important benefits of education (…), it can also be viewed as a powerful health policy which allows more people to enjoy both better and longer lives” [95].

## Figures and Tables

**Table 1:** Description of district-level indicators * investigated as potential predictors in the regression analyses: definitions, means, standard deviations (SD) and ranges; N = 401 German districts.

Indicator No.	Indicator Name	Definition [61,67]	Mean	SD	Range
(1)	Primary-care physicians per 100,000 inhabitants	Primary-care physicians (in German: “Hausärzte”) per 100,000 inhabitants	61.36	26.11	8.4–164.9
(2)	Hospital beds per 1000 inhabitants	Hospital beds per 1000 inhabitants	6.35	3.89	0.00–29.59
(3)	Care personnel per 100 persons in need of outpatient care services	Care personnel per 100 persons in need of outpatient care services	46.31	12.56	24.6–156.2
(4)	Care personnel per 100 persons in need of full inpatient care	Care personnel in nursing homes per 100 persons in need of full inpatient care	93.82	11.96	68.1–132.4
(5)	GDP (gross domestic product) per capita	GDP in 1000€ per inhabitant	37.09	16.05	16.4–172.4
(6)	Household income	Average disposable household income (in €) per inhabitant per month	1872.56	215.76	1365–3242
(7)	Proportion of employees without vocational qualification	Employees (subject to social security contributions) at place of residence without vocational qualification per 100 employees (subject to social security contributions) at place of residence	6.93	1.80	2.9–12.2
(8)	Proportion of employees with academic degree	Employees (subject to social insurance contributions) at place of residence with academic degree per 100 employees (subject to social security contributions) at place of residence	7.76	3.45	2.9–23.0
(9)	Unemployment	Unemployed or job-seeking persons per 1000 inhabitants at working age	44.24	19.22	12.2–106.3
(10)	Proportion of people with Hartz-IV support	Employable and non-employable persons entitled to German Social Code II-based welfare benefits (“Hartz-IV support”) per 100 inhabitants < 65 years	8.13	4.46	1.5–24.9
(11)	Proportion of elder with financial elder support	Persons > 64 years receiving basic income support per 1000 inhabitants > 64 years	22.37	14.63	3.0–82.1
(12)	Voter turnout	Voter turnout (in %) in the 2017 federal elections	75.08	3.79	63.1–84.1

* Sources: Indicators (1), (2) and (5)–(12) were taken from www.inkar.de (accessed on 5 December 2021) and reflect the year 2017 (exception: ‘hospital beds per 10,000 inhabitants’ dates from 2016) [58,60,61]. Regarding indicators (7)–(9), the numerators and denominators of these three indicators refer to people of working age which regularly ranges from 20 to 65 years of age. Indicators (3) and (4) were extracted from Regionaldatenbank Deutschland [62,67] and reflect the year 2017. Based on these district-level data, we, the authors, estimated means and standard deviations across districts.

**Table 2:** Results of bivariate regression analyses of men’s remaining life expectancy at age 60 at district level (dependent variable) with 12 potential predictors: ß (standardized regression coefficient) and *p* value of ß; N = 401 German districts.

Indicator/Predictor Name	ß (Standardized Regression Coefficient)	*p* Value of ß
Primary-care physicians per 100,000 inhabitants	−0.07	0.168
Hospital beds per 1000 inhabitants	−0.15	0.004
Care personnel per 100 persons in need of outpatient care services	0.14	0.005
Care personnel per 100 persons in need of full inpatient care	0.22	<0.001
GDP (gross domestic product) per capita	0.15	0.003
Household income	0.63	<0.001
Proportion of employees without vocational qualification	0.12	0.013
Proportion of employees with academic degree	0.41	<0.001
Unemployment	−0.60	<0.001
Proportion of people with Hartz-IV support	−0.56	<0.001
Proportion of elder with financial elder support	−0.10	0.045
Voter turnout	0.67	<0.001

**Table 3:** Results of bivariate regression analyses of women’s remaining life expectancy at age 60 at district level (dependent variable) with 12 potential predictors: ß (standardized regression coefficient) and *p* value of ß; N = 401 German districts.

Indicator/Predictor	ß (Standardized Regression Coefficient)	*p* Value of ß
Primary-care physicians per 100,000 inhabitants	−0.01	0.913
Hospital beds per 1000 inhabitants	−0.10	0.055
Care personnel per 100 persons in need of outpatient care services	0.14	0.005
Care personnel per 100 persons in need of full inpatient care	−0.00	0.981
GDP (gross domestic product) per capita	0.12	0.022
Household income	0.35	<0.001
Proportion of employees without vocational qualification	−0.14	0.006
Proportion of employees with academic degree	0.40	<0.001
Unemployment	−0.37	<0.001
Proportion of people with Hartz-IV support	−0.35	<0.001
Proportion of elder with financial elder support	−0.19	<0.001
Voter turnout	0.38	<0.001

**Table 4:** Results of a multiple regression analysis of men’s remaining life expectancy at age 60 at district level (dependent variable) and 11 potential predictors: results for potential predictors (ß; *p* value of ß); N = 401 German districts.

Indicator/Predictor	ß (Standardized Regression Coefficient)	*p* Value of ß
Primary-care physicians per 100,000 inhabitants	-	n.s.
Hospital beds per 1000 inhabitants	-	n.s.
Care personnel per 100 persons in need of outpatient care services	-	n.s.
Care personnel per 100 persons in need of full inpatient care	0.16	<0.001
GDP (gross domestic product) per capita	−0.13	0.005
Household income	0.27	<0.001
Proportion of employees without vocational qualification	-	n.s.
Proportion of employees with academic degree	0.42	<0.001
Unemployment	−0.23	<0.001
Proportion of elder with financial elder support	−0.09	0.050
Voter turnout	0.15	0.014

Explanations: n.s.—not significant.

**Table 5:** Results of a multiple regression analysis of women’s remaining life expectancy at age 60 at district level (dependent variable) and 11 potential predictors: results for potential predictors (ß; *p* value of ß); N = 401 German districts.

Indicator/Predictor	ß (Standardized Regression Coefficient)	*p* Value of ß
Primary-care physicians per 100,000 inhabitants	-	n.s.
Hospital beds per 1000 inhabitants	-	n.s.
Care personnel per 100 persons in need of outpatient care services	-	n.s.
Care personnel per 100 persons in need of full inpatient care	-	n.s.
GDP (gross domestic product) per capita	-	n.s.
Household income	-	n.s.
Proportion of employees without vocational qualification	-	n.s.
Proportion of employees with academic degree	0.51	<0.001
Unemployment	−0.23	<0.001
Proportion of elder with financial elder support	−0.30	<0.001
Voter turnout	-	n.s.

Explanations: n.s.—not significant.

## Data Availability

The data analyzed in this study were downloaded from the publicly available ‘INKAR’ and ‘Regionaldatenbank Deutschland’ databases [58,60,61,62,67].
